# Concurrent κ light chain proximal tubulopathy and crystal-storing histiocytosis in monoclonal gammopathy of renal significance: a rare case report with 4-year follow-up and literature review

**DOI:** 10.1186/s12882-026-05155-x

**Published:** 2026-07-15

**Authors:** Changxiu Guo, Rui Xue, Qinghui Zhou, Qijun Wan, Xiuli Zhang

**Affiliations:** 1 Department of Nephrolog, The First Affiliated Hospital of Shenzhen University (Shenzhen Second People’s Hospital), Shenzhen, China; 2 Department of Nephrology, Second People’s Hospital, The First Affiliated Hospital of Shenzhen University, Shenzhen, Guangdong Province 518000 P. R. China

**Keywords:** Light chain proximal tubulopathy, Crystal-storing histiocytosis, Monoclonal gammopathy of renal significance, Fanconi syndrome, Renal insufficiency, Chronic

## Abstract

**Background:**

Light chain proximal tubulopathy (LCPT) and crystal-storing histiocytosis (CSH) are rare forms of monoclonal immunoglobulin-associated kidney disease, representing either variants of monoclonal gammopathy of renal significance (MGRS) or renal complications of multiple myeloma (MM). Concurrent LCPT and suspected CSH associated with κ light chain MGRS is exceptionally rare, with only three comparable cases reported worldwide. This CARE guideline-compliant report describes the clinical, laboratory and histopathological features of this rare dual renal lesion.

**Case presentation:**

A 61-year-old Chinese woman presented with Fanconi syndrome (FS), chronic kidney disease (CKD) and isolated tubular proteinuria. Serum and urine immunofixation electrophoresis showed elevated monoclonal IgGκ. Bone marrow examination revealed mild plasmacytosis (< 10%), with no hypercalcemia, myeloma bone lesions, cast nephropathy or SLiM criteria-defined myeloma events, confirming MGRS and excluding MM and smoldering multiple myeloma (SMM).

**Diagnostic assessment:**

Renal biopsy electron microscopy demonstrated dense crystalline inclusions in proximal tubular epithelial cells and interstitial histiocytes. Immunohistochemistry and immunoelectron microscopy confirmed strong κ light chain restriction. These findings are highly suggestive of concurrent LCPT and crystal-storing histiocytosis (CSH). However, definitive confirmation of CSH by CD68 immunohistochemistry—the gold standard for histiocytic lineage identification—could not be performed due to exhaustion of the limited archival biopsy tissue. This case is therefore reported as “CSH suspected based on ultrastructural and immunoelectron microscopy criteria.

**Intervention and outcome:**

The patient received first-line CyBorD (cyclophosphamide, bortezomib, and dexamethasone) chemotherapy, achieving partial hematological remission (PR) and marked improvement of Fanconi syndrome. Renal function remained normal and stable throughout the 48-month follow-up period.

**Conclusions:**

We report this rare κ light chain MGRS case with concurrent LCPT and suspected CSH, which showed favorable responses to CyBorD with durable renal stability. This case highlights integrated diagnostic strategies and boosts clinical awareness of this rare monoclonal gammopathy-related renal injury.

**Supplementary Information:**

The online version contains supplementary material available at https://doi.org/10.1186/s12882-026-05155-x.

## Introduction

By the International Kidney and Monoclonal Gammopathy Research Group (IKMG) consensus, MGRS is defined as kidney injury caused by a small B-cell/plasma-cell clone not meeting MM/lymphoma criteria. Common morphological patterns of renal involvement include proliferative glomerulonephritis with monoclonal immunoglobulin deposits (PGNMID), monoclonal immunoglobulin deposition disease (MIDD), and amyloid light-chain (AL) amyloidosis [1, 2]. In rare instances, immunoglobulin crystallization occurs within proximal tubular cells (light chain proximal tubulopathy [LCPT]) [3] and histiocytes (crystal-storing histiocytosis [CSH]) [4]. In LCPT and CSH, the reabsorbed light chains are typically of the κ type, which possess inherent physicochemical properties that resist proteolysis and promote self-aggregation and crystal formation [5, 6]. LCPT is classified into crystalline and non-crystalline forms based on the presence or absence of crystalline structures within the cytoplasm of tubular epithelial cells [7, 8]. Crystal-storing histiocytosis (CSH) is a rare complication of multiple myeloma (MM) and other B-cell proliferative diseases. Furthermore, most of the reported cases were associated with multiple myeloma [9]. In patients with CSH, the bone marrow is the most commonly affected site, followed by lung/pleura, with the kidney being the third most common site of involvement [10]. Concurrent presentation of LCPT and CSH in the setting of MGRS is extremely rare, with limited cases documented globally, leading to under recognition and diagnostic delays in clinical practice. Herein, we report a rare case of MGRS secondary to monoclonal κ light chain, presenting with combined LCPT and suspected CSH accompanied by Fanconi syndrome and chronic kidney disease. Aiming to improve the clinical recognition, differential diagnosis, and management of this rare monoclonal gammopathy-related renal complication.

## Case presentation

A 61-year-old Chinese female was referred to our nephrology department for evaluation of progressive renal insufficiency and proteinuria. Baseline serum creatinine (SCr) was 131µmol/L (1.48 mg/dL). For the preceding three months, she had experienced polydipsia, polyuria, and nocturia, which had progressively worsened and substantially impaired her quality of life. She denied bone pain, fever, or rash. She had intermittently taken Chinese herbal medicine but had received no other therapeutic interventions.Fourteen days prior to admission, the patient was evalu-ated at another hospital, where laboratory tests revealed a SCr level of 177µmol/L (2.00 mg/dL). A monoclonal free κ spike was detected in in both urine and serum by immunofixation electrophoresis. She had abnormal serum free light chain (sFLC) levels: serum free κ light chain of 161.53 mg/L, free λ light chain of 17.46 mg/L, with a κ/λ ratio of 9.25. At admission, no significant medical history was documented, and physical examination revealed no specific abnormalities. Laboratory findings on admission were as follows (Supplementary Table 1). Laboratory studies revealed serum creatinine of 208.1µmol/L (2.35 mg/dL), uric acid of 162.7µmol/L, bicarbonate of 17.8 mmol/L, chloride of 113.2 mmol/L, potassium of 3.34 mmol/L, phosphate of 1.17 mmol/L. A morning urinalysis revealed 1⁺ protein, 2⁺ glucose, urine specific gravity of 1.006, and normal blood glucose. The 24-h urine protein was 1715.50 mg/day, albumin was 230 mg/day. A 24-hour urine phosphate level was 16.78 mmol/24 h, with a simultaneous serum phosphate of 1.17 mmol/L. The calculated fractional excretion of phosphate (FEp) was 39.17% (reference range < 20%), confirming marked renal phosphate wasting. The metabolic acidosis was of the normal anion gap type (serum anion gap 11.5 mmol/L), consistent with proximal renal tubular acidosis (type 2). Together with normoglycemic glycosuria, hypouricemia, hypokalemia, and low-molecular-weight proteinuria (predominantly tubular), these findings fulfilled the diagnostic criteria for Fanconi syndrome secondary to generalized proximal tubular dysfunction. Laboratory studies revealed serum β2-microglobulin of 4.92 mg/L, IgG of 1593 mg/dL, IgA of 204 mg/dL, IgM of 33 mg/dL, complement C3 of 153 mg/dL, and complement C4 of 41.8 mg/dL. Serum immunofixation electrophoresis demonstrated IgGκ M-protein positivity, with an M-protein level of 5.70 g/L. Serum free light chain analysis showed κ of 218.50 mg/L (reference, 3.30–19.40 mg/L), λ of 15.80 mg/L (reference, 5.71–26.3 mg/L), with a κ/λ ratio of 13.83 (reference, 0.26–1.65). Urinary free light chain analysis revealed κ of 270.0 mg/L (reference, 0.39–15.10 mg/L), λ of 47.50 mg/L (reference, 0.81–10.10 mg/L), with a κ/λ ratio of 5.68 (reference, 0.46-4.00). Bone marrow aspiration revealed 3.5% plasma cells, while flow cytometry analysis demonstrated 0.77% abnormal plasma cells with strong expression of CD38, CD138, and CD56. Bone marrow biopsy showed 8% plasma cells. The pathological diagnosis was consistent with clonal plasma cell proliferative disorder.

The patient presented with Fanconi syndrome (FS), as evidenced by normoglycemic glycosuria (urine glucose 2 + with normal blood glucose), hypouricemia (162.7 µmol/L), hypokalemia (3.34 mmol/L), renal phosphate wasting (FEp 39.17%, calculated from 24-hour urine phosphate of 16.78 mmol and simultaneous serum phosphate of 1.17 mmol/L), normal anion gap metabolic acidosis (serum bicarbonate 17.8 mmol/L, anion gap 11.5 mmol/L), and low-molecular-weight proteinuria (24-hour urine protein 1715.50 mg/day with only 230 mg/day albumin). Together with her symptoms of polydipsia and polyuria, these findings were diagnostic of Fanconi syndrome secondary to monoclonal κ light chain-mediated proximal tubular injury.

**Fig. 1:**
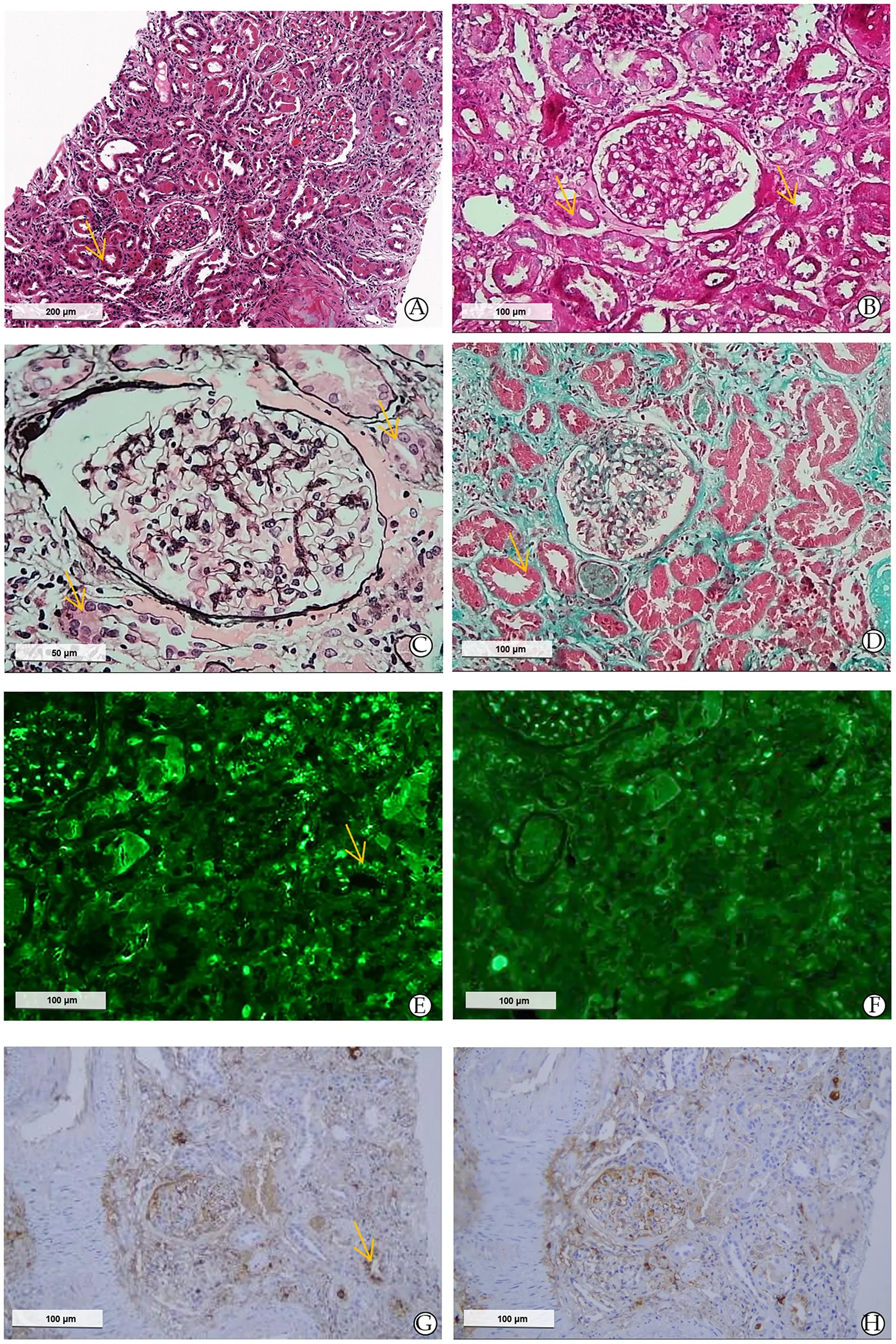
Findings on kidney biopsy. (**A**: HE staining, ×100; **B**: PAS staining, ×200; **C**: PASM staining, × 400; **D**: Masson staining, ×200)-Light microscopy revealed no significant alterations in glomeruli. Marked granular degeneration was observed in some proximal tubular epithelial cells, which contained intracellular strongly eosinophilic granules and lightly PAS-positive reabsorbed granular material. The renal interstitium exhibited multifocal inflammatory cell infiltration accompanied by mild fibrosis (arrows). (**E**: κ light chain,×200; **F**: λ light chain,×200)-Direct immunofluorescence of frozen tissue showed bright staining for κ light chain (arrows), while λ light chain was negative. (**G**: κ light chain,×200; **H**: λ light chain,×200)-Immunohistochemistry demonstrated κ light chain expression in proximal tubular epithelial cells (arrows), whereas λ light chain was negative

**Fig. 2:**
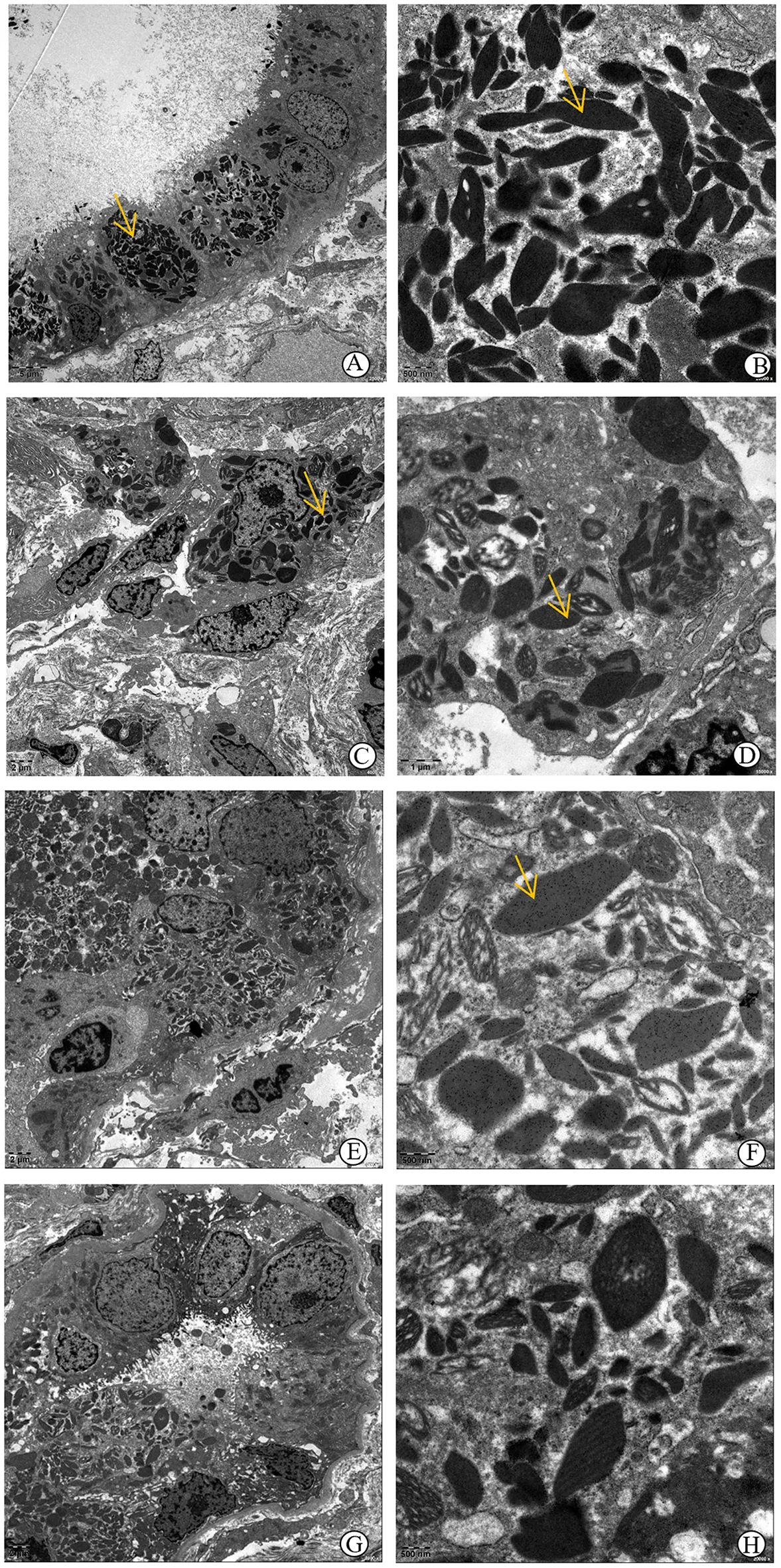
Electron microscopy and immunoelectron microscopy findings. (**A**, **B**) Proximal tubular epithelial cells containing abundant intracytoplasmic crystalline inclusions of varying geometric shapes, including rhomboid, rectangular, and needle-like configurations (arrows) (**A**: original magnification ×2,500, bar = 5 μm; **B**: ×20,000, bar = 500 nm). (**C**, **D**) Interstitial infiltration by large polygonal cells with abundant cytoplasm, eccentric reniform nuclei, and prominent phagolysosomal compartments, morphologically consistent with histiocytes/macrophages. These cells contain numerous intracytoplasmic crystalline inclusions identical to those seen in proximal tubular epithelium (arrows) (**C**: ×4,000, bar = 2 μm; **D**: ×15,000, bar = 1 μm). Note the absence of epithelial junctional complexes and basement membrane attachment, excluding tubular epithelial origin. (**E**, **F**) Immunoelectron microscopy demonstrating intense gold labeling for κ light chain localized specifically to the crystalline inclusions within both proximal tubular epithelial cells and the interstitial histiocytes. Note the dense gold particle deposition at ×25,000 magnification (arrows) (**E**: ×4,000, bar = 2 μm; **F**: ×25,000, bar = 500 nm). (**G**, **H**) Corresponding immunoelectron microscopy for λ light chain showing negative staining in the same cellular compartments, confirming κ light chain restriction (**G**: ×4,000, bar = 2 μm; **H**: ×20,000, bar = 500 nm)

A renal biopsy was subsequently performed. Light microscopy examination revealed 18 glomeruli, of which 2 showed global sclerosis. Significant tubular alterations were observed, characterized by prominent proximal tubular epithelial cells exhibiting severe granular degeneration with intensely eosinophilic granules and abundant PAS-positive reabsorption droplets. Chronic tubulointerstitial changes were mild, with approximately 5–10% of cortical tubules showing atrophy, multifocal interstitial inflammatory cell infiltration involving less than 25% of the cortical interstitium, and mild interstitial fibrosis (interstitial fibrosis/tubular atrophy, IFTA, graded as < 25%) (Fig. 1A-D). Direct immunofluorescence of frozen tissue (IF-F) revealed that there was no specific staining of IgG, IgA, IgM, C3, and λ light chain on glomeruli, tubular epithelial cells, interstitium, and vessels. However, specific staining for κ light chain was observed within the proximal tubular epithelial cells (Fig. 1E, F). Immunohistochemical staining revealed that the proximal tubular epithelial cells were positive for κ light chain but negative for λ light chain (Fig. 1G, H). Congo red staining was not performed; however, no amorphous eosinophilic extracellular deposits suggestive of amyloid were observed on light microscopy, and electron microscopy revealed no randomly oriented fibrils (8–12 nm in diameter) characteristic of amyloid deposition. These findings effectively exclude concurrent AL amyloidosis. Electron microscopy revealed no electron-dense deposits in glomeruli. However, numerous dense crystalline inclusions of varied shapes were identified within proximal tubular epithelial cells (Fig. 2A, B). Notably, the interstitial compartment contained large polygonal cells with ultrastructural features of histiocytes—abundant cytoplasm, eccentric nuclei, and prominent lysosomes—harboring identical crystalline inclusions (Fig. 2C, D). Immunoelectron microscopy demonstrated intense positivity for κ light chain (Fig. 2E, F) in both the proximal tubular epithelial cells and these interstitial histiocytes, while λ light chain (Fig. 2G, H) was negative. Notably, the original pathology report from the reference laboratory (Guangzhou Kingmed Center) explicitly identified the interstitial crystal-laden cells as histiocytes based on ultrastructural features, and rendered a final diagnostic conclusion of crystal-storing histiocytosis. These findings are highly suggestive of concurrent LCPT and CSH. The interstitial crystal-laden cells exhibited ultrastructural features of histiocytes (abundant cytoplasm, eccentric reniform nuclei, prominent phagolysosomes, absence of epithelial junctions), and immunoelectron microscopy confirmed κ light chain restriction within these cells. However, the gold standard for CSH diagnosis—demonstration of κ light chain co-localization with CD68-positive histiocytes by double immunostaining or serial section analysis—could not be performed because the archival renal biopsy tissue obtained in 2021 was completely exhausted during the initial diagnostic workup. Therefore, while the findings are strongly suggestive of CSH, we report this case as “CSH suspected” and acknowledge this as a major limitation.

### Diagnosis

**Fig. 3:**
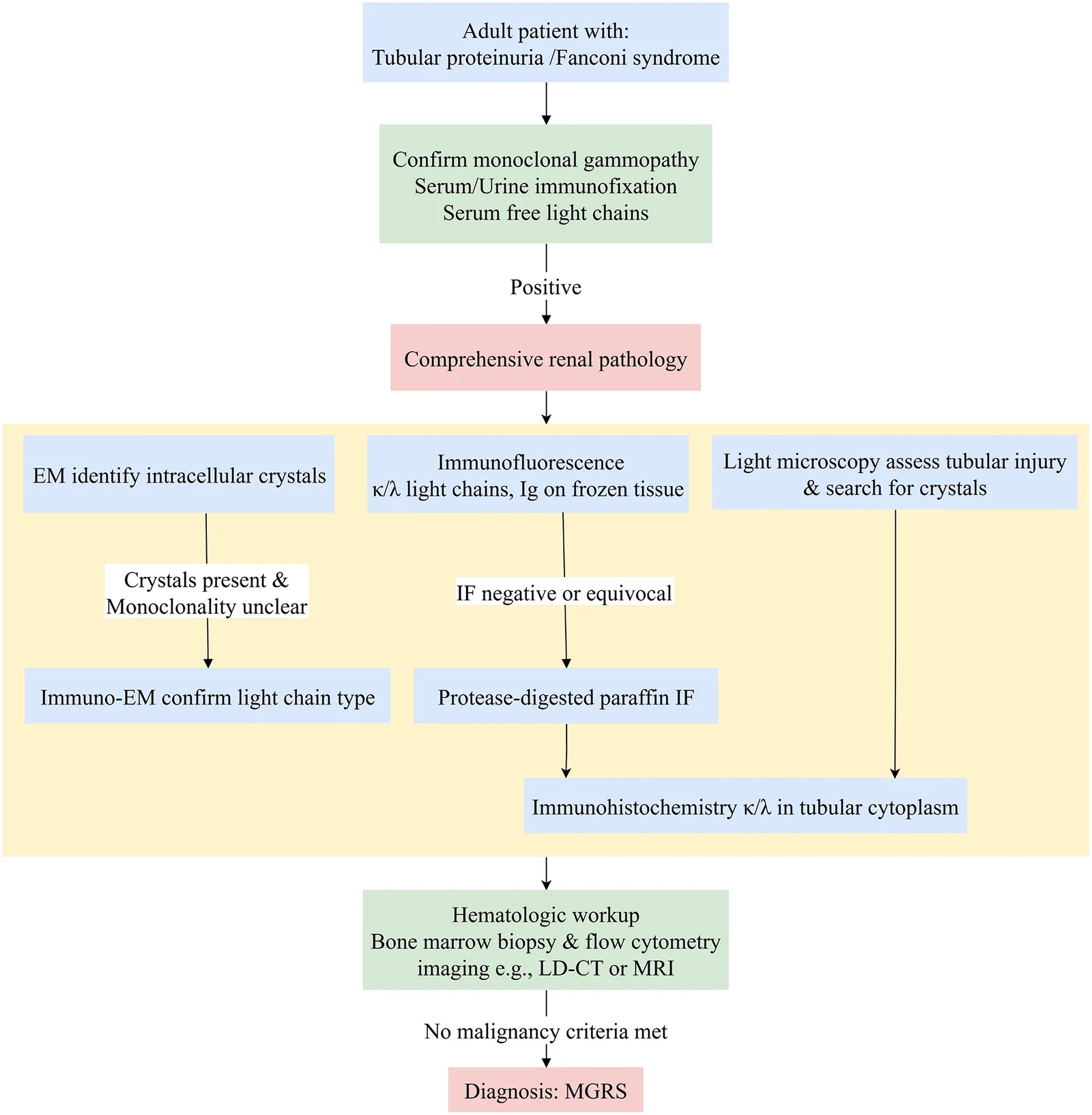
Diagnostic algorithm for MGRS presenting with tubular proteinuria or FS. The diagnostic process is triggered in an adult patient with unexplained tubular proteinuria and/or FS. The initial step is to confirm the presence of a monoclonal protein. If positive, a kidney biopsy is the cornerstone of diagnosis. A comprehensive pathological evaluation is essential, including light microscopy to identify tubular injury and crystals, standard IF, and EM to detect crystalline inclusions. If IF on frozen tissue is negative or equivocal, protease-digested paraffin IF is a critical subsequent step with high sensitivity for detecting LCPT and CSH. IHC can demonstrate light chain restriction within tubular epithelial cells. When crystals are identified by EM but monotypia is not confirmed by IF or IHC, immuno-EM serves as the gold standard to confirm the light chain type of the crystals. Following histologic confirmation, a hematologic workup is mandatory to rule out a malignant lymphoproliferative disorder. If no malignancy is found, a diagnosis of MGRS is established. MGRS, monoclonal gammopathy of renal significance; FS, fanconi syndrome; LCPT, light chain proximal tubulopathy; CSH, crystal-storing histiocytosis; IF, immunofluorescence; EM, electron microscopy; IHC, Immuno-histochemistry; Immuno-EM, Immunoelectron Microscopy; LD-CT, low-dose whole-body computed tomog-raphy; MRI; magnetic resonance imaging

Serum and urine analyses revealed a monoclonal immunoglobulin (M protein) and an abnormal κ/λ free light-chain ratio. Renal biopsy demonstrated monoclonal immunoglobulin–associated renal lesions, whereas bone marrow aspiration and biopsy confirmed clonal plasma cells that remained below the 10% threshold required for a diagnosis of multiple myeloma. In addition, only anemia was present; no CRB features (hypercalcemia, renal insufficiency, or bone lesions) were present. Importantly, although renal impairment was documented, kidney biopsy demonstrated LCPT rather than myeloma cast nephropathy, and the hematologic burden did not meet diagnostic criteria for MM. And none of the SLiM criteria (≥ 60% bone marrow plasma cells, serum free light-chain ratio ≥ 100, or > 1 focal osseous lesion on MRI) were met. Consequently, diagnoses of MM or SMM was excluded [11]. In accordance with the IKMG consensus [2], this case fulfills the criteria for MGRS, with renal histopathology characterized by monoclonal κ light chain deposition in proximal tubular epithelial cells and interstitial histiocytes. Clinically, in patients presenting with monoclonal gammopathy accompanied by tubular proteinuria and/or FS, kidney biopsy should be strongly recommended, a strategy fully aligned with the core recommendations of the IKMG consensus [2]. Our recommended approach to renal biopsy analysis in patients suspected to have MGRS is provided in Fig. 3.

## Treatment and outcome

**Fig. 4:**
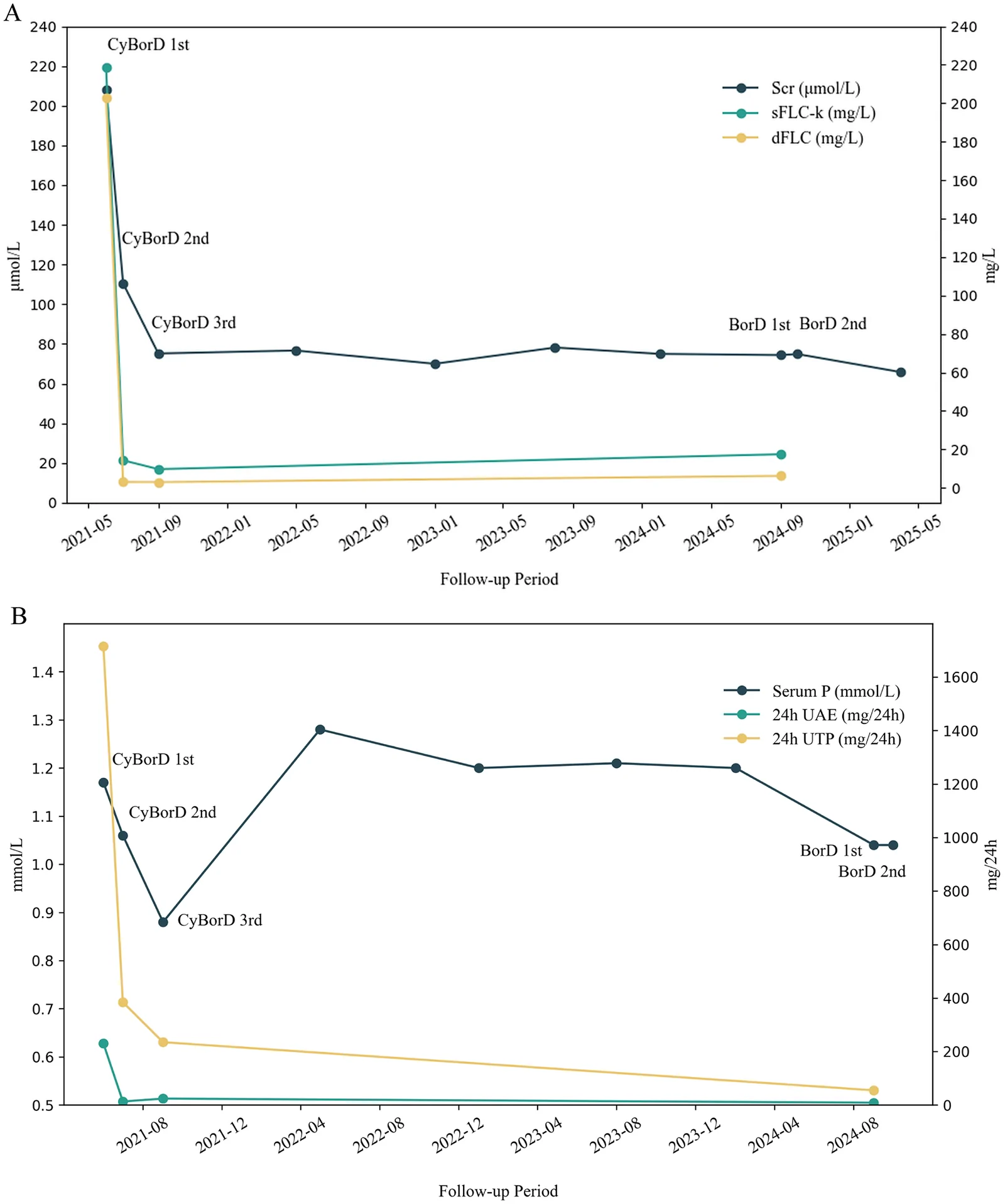
**A**. Changes in Scr and sFLC-κ levels over follow-up. The patient received 3 cycles of CyBorD from June 2021 to September 2021, followed by 2 cycles of BorD in September–October 2024. CyBorD cyclophosphamide, bortezomib, and dexamethasone, BorD bortezomib and dexamethasone, sFLC serum free light chain, dFLC the difference between involved and uninvolved sFLC, sFLC-κ serum free κ light chain, SCr serum creatinine (1 mg/dL = 88.4 μmol/L). **B**. Changes in serum P and tubular proteinuria levels over follow-up. CyBorD cyclophosphamide, bortezomib, and dexamethasone, BorD bortezomib and dexamethasone, 24h UAE, 24-hour urinary albumin excretion, 24h UTP 24-h urinary total protein

The patient consented to receive first-line CyBorD (cyclophosphamide, bortezomib, and dexamethasone) combination chemotherapy for the clonal plasma cell disorder. For treatment tolerability: The patient tolerated the CyBorD regimen excellently. No treatment-emergent adverse events of clinical significance occurred. Specifically, there was no evidence of bortezomib-induced peripheral neuropathy, cyclophosphamide-related cystitis or myelosuppression, dexamethasone-associated hyperglycemia or gastrointestinal discomfort. Vital signs remained stable, and no fever, infection, hypotension or rash was noted during chemotherapy. All planned cycles were completed as scheduled, with no dose adjustment, pause or termination of treatment.For therapeutic efficacy: After one cycle of CyBorD, obvious improvements were observed in both hematological parameters and renal manifestations. Serum immunofixation electrophoresis remained positive for IgGκ M-protein, while the M-protein level decreased from 5.70 g/L to 2.20 g/L. Serum free κ light chain declined sharply from 218.50 mg/L to 14.28 mg/L, and the κ/λ ratio returned to the normal range (1.30). The 24-hour total urinary protein excretion dropped markedly from 1715.50 mg/day to 384.56 mg/day. Serum creatinine decreased from 208.1 µmol/L (2.35 mg/dL) to 110.5 µmol/L (1.25 mg/dL), indicating significant recovery of renal function. Meanwhile, all clinical manifestations of Fanconi syndrome, including normoglycemic glycosuria, renal phosphate wasting, hypokalemia and metabolic acidosis, were completely resolved, accompanied by relief of polydipsia, polyuria and nocturia. The patient therefore achieved partial hematological remission (PR). Given mild bilateral lower extremity edema at the 39-month follow-up, two additional cycles of BorD (bortezomib plus dexamethasone) were administered. Similarly, the patient tolerated the BorD regimen well, with no new adverse reactions. Over the total 48-month follow-up period, serum creatinine remained stably maintained between 65.8 µmol/L (0.74 mg/dL) and 110.5 µmol/L (1.25 mg/dL), and hematological indicators stayed within a stable range, demonstrating durable hematological and renal responses to bortezomib-based therapy (Fig. 4A, B). The timeline of key events was shown in Supplementary Table 2. 

Changes in Scr and sFLC-κ levels over follow-up. The patient received 3 cycles of CyBorD from June 2021 to September 2021, followed by 2 cycles of BorD in September–October 2024. CyBorD cyclophosphamide, bortezomib, and dexamethasone, BorD bortezomib and dexamethasone, sFLC serum free light chain, dFLC the difference between involved and uninvolved sFLC, sFLC-κ serum free κ light chain, SCr serum creatinine (1 mg/dL = 88.4 µmol/L).

Changes in serum P and tubular proteinuria levels over follow-up. CyBorD cyclophosphamide, bortezomib, and dexamethasone, BorD bortezomib and dexamethasone, 24 h UAE, 24-hour urinary albumin excretion, 24 h UTP 24-h urinary total protein.

## Patient perspective

Prior to diagnosis, the patient had experienced progressive symptoms of polydipsia and polyuria that substantially compromised her well-being and quality of life. Although she and her family were initially concerned about the diagnosis of a rare kidney disorder (MGRS) and the need for chemotherapy, thorough counseling helped her understand the necessity of clone-directed therapy to preserve renal function. She subsequently provided informed consent for the CyBorD regimen. The patient tolerated the treatment well. During the long-term follow-up, she has expressed strong satisfaction with her treatment outcomes, particularly the stabilization of her kidney function over four years, which has allowed her to maintain a normal lifestyle. She remains engaged in her regular follow-up appointments, understanding the importance of ongoing monitoring.

## Literature review

To comprehensively understand the reported cases of concurrent LCPT and CSH. we performed a systematic PubMed search with the term ‘light chain proximal tubulopathy or crystal-storing histiocytosis’, and identified 294 relevant articles from 1978 to June 2025. Titles and abstracts of the retrieved records were screened, and full-text reviews were performed to confirm pathological diagnoses documenting both LCPT and CSH. We identified 12 reported cases with well-documented co-occurrence of LCPT and CSH. Further analysis revealed that the majority of these cases were associated with MM. Notably, after excluding myeloma-related cases, only three cases were attributed to MGRS. We therefore summarized the clinical data of these four MGRS-related cases—including the present one—covering age, sex, serum creatinine, urine protein, clinical manifestations, monoclonal protein type, hematologic disorder, crystal distribution, treatment, and outcomes (Supplementary Table 3).

## Discussion

We report a rare case of MGRS manifesting as combined LCPT and suspected CSH. The case exemplifies the core principle of MGRS: a clonal disorder, which itself may be clinically silent or benign by hematologic standards (as evidenced by < 10% bone marrow plasma cells and no MDEs), can produce a nephrotoxic monoclonal immunoglobulin that leads to significant renal pathology. Notably, our patient achieved sustained PR with bortezomib-based chemotherapy, providing valuable insights into the management of this rare condition.

Recent insights into LCPT reveal a complex pathogenesis and varied clinical presentations. LCPT commonly presents with FS, tubular proteinuria, and varying degrees of renal impairment [12, 13]. The pathogenesis of LCPT involves a sophisticated interplay between molecular structure and cellular processing. The monoclonal free light chains (FLCs) filtered through the glomerulus can be reabsorbed through the megalin/cubilin complex on the luminal surface of the proximal tubule cells. However, certain FLCs resist lysosomal degradation and accumulate intracellularly, leading to tubular injury [6]. Histologically, LCPT manifests in two main forms: crystalline and non-crystalline. The crystalline type, as observed in our patient, features geometric inclusions such as rhomboid, rectangular, or needle-like structures, while non-crystalline deposits appear as granular or vacuolated changes [14, 15]. CSH involves accumulation of histiocytes containing immunoglobulin crystals, primarily in the bone marrow, with renal involvement reported as the third most common site after bone marrow and lung/pleura [10]. In the kidney CSH is rarely a stand-alone pathology, and is in fact, often found co- existing with other morphologic forms of monoclonal renal disease, including LCPT, crystalloid glomerulopathy or cast nephropathy, suggesting that they may share similar pathogenic mechanisms [4, 16, 17].Consistent with our case, both LCPT and CSH are predominantly associated with monoclonal κ light chains, supporting the pivotal role of light chain type in crystal formation [11, 18]. Researchers have proposed that specific mutations in the variable region of monoclonal κ light chains lead to alterations in amino acid composition, specifically the substitution of a polar serine residue by a non-polar hydrophobic amino acid residue. This structural modification renders the κ light chains resistant to normal degradation by lysosomal enzymes, thereby facilitating the formation of regularly arranged crystalline structures within the endolysosomal compartment under particular microenvironmental conditions [19]. In this case, immunoelectron microscopy revealed κ light chain deposits with similar crystalline morphology in both proximal tubular cells and interstitial histiocytes, providing morphological evidence for potentially shared pathogenic mechanisms between LCPT and CSH.

Regarding the pathological diagnosis of renal CSH in this case. The gold standard for diagnosing renal crystal-storing histiocytosis requires demonstration of monotypic light chain restriction within CD68-positive histiocytes, typically achieved by double immunostaining (CD68/κ) or serial section analysis [4, 10]. In the present case, while ultrastructural examination revealed cells with unequivocal histiocytic morphology (abundant cytoplasm, eccentric reniform nuclei, prominent phagolysosomal compartments, absence of epithelial junctional complexes), and immunoelectron microscopy confirmed κ light chain restriction within the crystalline inclusions of these cells, we were unable to perform CD68 immunohistochemistry due to complete exhaustion of the archival biopsy tissue. Some published case series of renal CSH have accepted the combination of characteristic ultrastructure and immuno-EM-based light chain restriction as sufficient for diagnosis in retrospective settings where tissue is limited [4]. Nevertheless, we recognize the absence of CD68 co-localization as a significant limitation. Accordingly, we have described this case as “CSH strongly suspected” rather than definitively proven throughout this report. We encourage future prospective studies to incorporate CD68/κ double staining as a mandatory diagnostic criterion for renal CSH.

According to the IKMG research group consensus, the diagnosis of MGRS-associated LCPT and CSH in patients with unexplained kidney disease and a monoclonal gammopathy requires both a renal biopsy and a comprehensive hematologic workup. Renal pathological evaluation should include light microscopy, immunofluorescence, and electron microscopy [2]. Particularly, proteinuria ≥ 1.5 g/d, hematuria, and an elevated free light chain ratio increase the likelihood of finding MGRS [20], and a kidney biopsy should be highly considered in such patients. Treatment should be initiated promptly once renal injury is attributable to the monoclonal immunoglobulin, particularly in MGRS patients presenting with progressive renal deterioration or significant proteinuria. The primary therapeutic objective is the eradication of the abnormal plasma cell or B-cell clone responsible for producing the pathogenic monoclonal immunoglobulin. Bortezomib-based chemotherapeutic regimens or CD38-targeted monoclonal antibodies (e.g., daratumumab) represent a reasonable and effective first-line choice [21, 22].Supporting this, a recent case report demonstrated that in a patient with LCPT unresponsive to lenalidomide, a daratumumab-based regimen (followed by Dara-CyborD) achieved complete hematologic remission, normalized serum potassium, and reduced serum creatinine by 50%, further validating the efficacy of CD38-targeted therapy in LCPT [23]. In the present case, administration of the CyBorD regimen (cyclophosphamide, bortezomib, and dexamethasone) led to a rapid and sustained partial remission, which aligns with previous reports indicating that treatment can significantly improve renal outcomes in patients with LCPT [14, 15]. In contrast to three previously reported cases of MGRS-associated LCPT with concurrent CSH, our patient achieved the most rapid remission and maintained a sustained response during long-term follow-up (Supplementary Table 3). Supporting this strategy, a documented case of MGRS-associated LCPT/CSH achieved a very good partial remission (VGPR) following daratumumab therapy after an insufficient response to CyBorD [9]. For eligible patients, autologous stem cell transplantation (ASCT) remains a potent consolidation option, with evidence from several LCPT case series demonstrating its capacity to induce long-term responses [15].

Comparison with previously reported cases. A recent case by Zhu L et al. described combined LCPT, CSH, and crystalline podocytopathy in a patient with multiple myeloma (MM) [24]. In contrast, our patient fulfilled the diagnostic criteria for MGRS rather than MM, as evidenced by bone marrow plasma cells < 10%, absence of myeloma-defining events, and no CRAB/SLiM criteria. This distinction is clinically significant because patients with MGRS require clone-directed therapy primarily for renal protection rather than for malignancy control. Moreover, our case provides the longest follow-up duration (48 months) among the four reported MGRS-associated LCPT/CSH cases, demonstrating the durability of renal response to bortezomib-based therapy. These features underscore the novelty and clinical relevance of our report.

### Strengths and Limitations

The strengths of our study include the comprehensive pathological workup with advanced techniques like immunoelectron microscopy, the 48‑month long‑term follow‑up demonstrating durable treatment response, and the detailed documentation of this exceedingly rare dual pathology. The diagnostic algorithm we propose (Fig. 3) may assist clinicians in approaching similar cases. However, several limitations must be acknowledged. First and most importantly, the diagnosis of CSH in this case lacks CD68 immunohistochemical confirmation—the current gold standard for histiocytic lineage identification. While we provide ultrastructural evidence of histiocyte morphology and κ light chain restriction by immunoelectron microscopy, the inability to perform CD68/κ double staining or serial section analysis means that the diagnosis of CSH remains presumptive. We have therefore described this case as “suspected CSH” throughout the manuscript. Second, Congo red staining was not performed to exclude amyloidosis, though no histological or ultrastructural evidence of amyloid (amorphous eosinophilic deposits on light microscopy or fibrils on electron microscopy) was identified. Third, as a retrospective case report relying on archived pathology material from 2021, the resolution of the immunoelectron microscopy images is suboptimal; however, the κ light chain restriction was independently confirmed by the reference laboratory’s certified pathology report. Fourth, urinary amino acid excretion was not measured, and longitudinal serum bicarbonate data were unavailable during follow‑up; however, the normalization of serum phosphate and resolution of tubular proteinuria serve as robust surrogate markers for the resolution of Fanconi syndrome. Fifth, as a single case report, our conclusions are inherently limited and require validation through larger, multi‑center collaborative studies.

## Conclusions

In summary, when a patient presents with proximal tubulopathy and a detectable monoclonal immunoglobulin, concurrent bone marrow and renal biopsies are strongly recommended to establish a definitive diagnosis. Once MGRS is confirmed, clone-directed therapy based on bortezomib or anti-CD38 regimens should be initiated promptly according to the patient’s renal manifestations. For eligible patients, ASCT should be considered to prevent further renal deterioration. Enhancing clinicians’ awareness of this diagnostic and therapeutic pathway is crucial for improving patient outcomes.

## Supplementary Information

Below is the link to the electronic supplementary material.

Supplementary Material 1

Supplementary Material 2

Supplementary Material 3

## Data Availability

The data that support the findings of this case report are available from the corresponding author upon reasonable request. All relevant clinical and laboratory data have been included in the present manuscript and its associated tables/figures.

## Abbreviations

ASCT: Autologous stem cell transplantation
BorD: Bortezomib and dexamethasone
CKD: Chronic kidney disease
CSH: Crystal-storing histiocytosis
CyBorD: Cyclophosphamide, bortezomib, and dexamethasone
dFLC: difference between involved and uninvolved serum free light chain
EM: Electron microscopy
FS: Fanconi syndrome
IF: Immunofluorescence
IHC: Immunohistochemistry
IKMG: International Kidney and Monoclonal Gammopathy Research Group
LCPT: Light chain proximal tubulopathy
MGRS: Monoclonal gammopathy of renal significance
MM: Multiple myeloma
PAS: Periodic acid–Schiff
PR: Partial remission
SCr: Serum creatinine
sFLC: Serum free light chain
VGPR: Very good partial remission
