# Supplementary material for: Concurrent κ light chain proximal tubulopathy and crystal-storing histiocytosis in monoclonal gammopathy of renal significance: a rare case report with 4-year follow-up and literature review
Source: BMC Nephrol. 2026 Jul 15;27:604. doi: 10.1186/s12882-026-05155-x (PMC13656593; doi:10.1186/s12882-026-05155-x)
Supplement: Supplementary file 1 [file 12882_2026_5155_MOESM1_ESM.docx]

*Supplementary Material*

**Concurrent κ light chain proximal tubulopathy and crystal-storing histiocytosis in monoclonal gammopathy of renal significance: a rare case report with 4-year follow-up and literature review**

***Changxiu Guo, Rui Xue, Qinghui Zhou, Qijun Wan, Xiuli Zhang****

**Correspondence authors: Xiuli Zhang:* [*Zhang2013_12@163.com*](mailto:Zhang2013_12@163.com)

**Supplementary Table 1: Blood and urine test results**

| Variable | Lab value | Reference Range |
| --- | --- | --- |
| Peripheral blood |  |  |
| WBC(×10⁹/L) | 7.71 | 3.50-9.50 |
| RBC (×10¹²/L) | 3.78 | 3.8-5.1 |
| Hb(g/L) | 110.1 | 115-150 |
| Plt(×10⁹/L) | 357.0 | 125-350 |
| Urinalysis |  |  |
| Specific gravity | 1.005 | 1.003-1.030 |
| pH | 7.0 | 4.5-8.0 |
| Protein | (1+) | Negative |
| Occult blood | (1+) | Negative |
| Glucose | (2+) | Negative |
| Ketone | Negative | Negative |
| β2MG(mg/L） | 7.68 | Negative |
| RBC（/HPF） | 20.6 | 0-4 |
| WBC（/HPF） | 3.96 | 0-5 |
| Hyaline Cast（/HPF） | 0 | 0-3 |
| Granular Cast（/HPF） | 0 | 0-3 |
| 24-hour urine protein (mg/24h) | 1715.50 | 0-150 |
| 24-hour urinary albumin excretion (mg/24h) | 230.00 | 0-30 |
| Blood Chemistry |  |  |
| TP （g/L) | 74.0 | 65.0-85.0 |
| Alb（g/L) | 40.6 | 40.0-55.0 |
| AST (U/L) | 11 | 13-35 |
| ALT (U/L) | 11.9 | 7-40 |
| ALP (U/L) | 56 | 50-135 |
| LDH (U/L) | 181 | 120-250 |
| TC mmol/L) | 5.24 | <5.2 |
| TG (mmol/L) | 2.98 | <1.7 |
| Na (mmol/L) | 142.5 | 137-147 |
| K (mmol/L) | 3.34 | 3.5-5.3 |
| Cl (mmol/L) | 113.2 | 99-110 |
| Ca (mmol/L) | 2.19 | 2.11-2.52 |
| P (mmol/L) | 1.17 | 0.85-1.51 |
| BUN (mmol/L) | 6.2 | 3.1-8.8 |
| Cre (mol/L) | 208.1 | 41-81 |
| UA (μmol/L) | 165.2 | 155-357 |
| HCO3⁻ (mmol/L) | 17.8 | 20.0-30.0 |
| %TRP (%) | 60.84 | 85-95 |
| Glu(mmol/L) | 5.16 | 3.89-6.11 |
| β2MG (mg/L) | 4.92 | 0.8-1.8 |
| Blood Coagulation Test |  |  |
| PT (sec) | 11.2 | 9.8-12.1 |
| APTT (sec) | 23.9 | 24-35 |
| Fib (g/L) | 5.49 | 2.0-4.0 |
| D-D (mg/L) | 0.15 | <0.55 |
| Immunological Findings |  |  |
| %M-protein (%) | 7.50 | not available |
| M-protein (g/L) | 5.70 | not available |
| IgG (mg/dL) | 1593 | 700-1600 |
| IgA (mg/dL) | 204 | 70-400 |
| IgM (mg/dL) | 33 | 40-230 |
| C3 (mg/dL) | 153 | 90-180 |
| C4(mg/dL) | 41.8 | 10-40 |
| CRP (mg/L) | 4.19 | 0-5 |
| HBsAg（IU/ml） | 0.00 | 0-0.03 |
| HCV Ab（C.O.I） | 0.00 | 0-1 |
| TP-Ab （C.O.I） | 0.00 | 0-1 |
| HIV-Ab （C.O.I） | 0.00 | 0-1 |
| MPO–ANCA | Negative | Negative |
| PR3-ANCA | Negative | Negative |
| AntiGBM Ab | Negative | Negative |
| ANA | Negative | Negative |
| ENA | Negative | Negative |
| Serum Free Light Chain |  |  |
| κ(mg/L) | 218.50 | 3.30-19.40 |
| λ(mg/L) | 15.80 | 5.71-26.30 |
| κ/λ ratio | 13.83 | 0.26-1.65 |
| dFLC(mg/L) | 202.70 | not available |
| Urinary Free Light Chain |  |  |
| κ(mg/L) | 270.00 | 0.39-15.10 |
| λ(mg/L) | 47.50 | 0.81-10.10 |
| κ/λ ratio | 5.68 | 0.46-4.00 |
| dFLC(mg/L) | 222.50 | not available |

Abbreviation: CRP: C-reactive protein; C3, C4: complement proteins C3, C4; ANA: antinuclear antibodies; PR3-ANCA: anti-proteinase 3 antineutrophil cytoplasmic antibody; MPO-ANCA: anti- myeloperoxidase antineutrophil cytoplasmic antibody; %TRP: %Tubular Reabsorption of Phosphate; dFLC: the difference between involved and uninvolved sFLC; TC, total cholesterol; TG, triglycerides; sFLC, serum free light chain; Hb, hemoglobin; ALB, albumin; ALT, alanine aminotransferase; AST, aspartate aminotransferase.
