# Supplementary material for: Concurrent κ light chain proximal tubulopathy and crystal-storing histiocytosis in monoclonal gammopathy of renal significance: a rare case report with 4-year follow-up and literature review
Source: BMC Nephrol. 2026 Jul 15;27:604. doi: 10.1186/s12882-026-05155-x (PMC13656593; doi:10.1186/s12882-026-05155-x)
Supplement: Supplementary file 2 [file 12882_2026_5155_MOESM2_ESM.docx]

**Supplementary Material**

**Concurrent κ light chain proximal tubulopathy and crystal-storing histiocytosis in monoclonal gammopathy of renal significance: a rare case report with 4-year follow-up and literature review**

***Changxiu Guo, Rui Xue, Qinghui Zhou, Qijun Wan, Xiuli Zhang****

**Correspondence authors: Xiuli Zhang:* [*Zhang2013_12@163.com*](mailto:Zhang2013_12@163.com)

**Supplementary Table 2: Key clinical timeline according to CARE guidelines**

| Time point | Event |
| --- | --- |
| March 2021 | Onset of polydipsia, polyuria, and nocturia |
| June 18, 2021 | Kidney biopsy performed |
| June 2021 | Initiation of CyBorD cycle 1 |
| July 2021 | CyBorD cycle 2 |
| September 2021 | Completion of CyBorD cycle 3; partial hematological remission achieved |
| May 2022 | Follow-up: SCr 76.7 μmol/L |
| September 2024 | Initiation of BorD cycle 1 (due to mild edema and increased dFLC) |
| October 2024 | Completion of BorD cycle 2 |
| April 2025 | Last follow-up: SCr 65.8 μmol/L |

Abbreviation: CyBorD: cyclophosphamide, bortezomib and dexamethasone; BorD: bortezomib and dexamethasone; dFLC : the difference between involved and uninvolved sFLC; sFLC: serum free light chain; SCr: serum creatinine
