# Supplementary material for: Concurrent κ light chain proximal tubulopathy and crystal-storing histiocytosis in monoclonal gammopathy of renal significance: a rare case report with 4-year follow-up and literature review
Source: BMC Nephrol. 2026 Jul 15;27:604. doi: 10.1186/s12882-026-05155-x (PMC13656593; doi:10.1186/s12882-026-05155-x)
Supplement: Supplementary file 3 [file 12882_2026_5155_MOESM3_ESM.docx]

*Supplementary Material*

**Concurrent κ light chain proximal tubulopathy and crystal-storing histiocytosis in monoclonal gammopathy of renal significance: a rare case report with 4-year follow-up and literature review**

***Changxiu Guo, Rui Xue, Qinghui Zhou, Qijun Wan, Xiuli Zhang****

**Correspondence authors: Xiuli Zhang:* [*Zhang2013_12@163.com*](mailto:Zhang2013_12@163.com)

**Supplementary table 3: Previous reports of cases with combined LCPT and CSH cauesd by MGRS or MGUS**

| **Author[Ref]** | **Age/sex** | **Serum Creatinine (Scr, mg/dL)** | **Urine Protein (g/day)** | **Clinical Renal Manifestation** | **Plasma cell dyscrasia** | **Hematologic Disease** | **Crystal Distribution** | **Treatment** | **Prognosis** | **Follow-up period（months）** |
| --- | --- | --- | --- | --- | --- | --- | --- | --- | --- | --- |
| Qin et al. [S1] | 67/M | 10.45→1.81 | 2.01 | AKI, partial Fanconi syndrome | κ light chain | MGRS | Proximal tubular epithelial cells, renal interstitial histiocytes, bone marrow histiocytes | 4 cycles of CyBorD, 1 cycle of BorD combined with daratumumab and one more cycle of BorD | VGPR, stable renal function(SCr1.4→1.6mg/dl), improved Fanconi syndrome | 30 |
| Nakamura et al. [S2] | 66/F | 3.32 → N/A | 2.20 | CKD | Bence–Jones κ | MGRS | Proximal tubular epithelial cells, renal interstitial histiocytes | NA | NA | NA |
| Ito et al. [S3] | 65/F | 1.94 → eGFR 36mL/min/1.73m² | 1.31 | CKD without Fanconi syndrome | IgGκ | MGUS | Podocytes, tubular epithelial cells, renal interstitial histiocytes | 9 courses of bortezomib and dexamethasone | Renal function improved to eGFR 36 mL/min/1.73m² | 10 |
| Present case | 61/F | 2.35→0.74 | 1.72 | CKDwithFanconi syndrome | IgGκ | MGRS | Proximal tubular epithelial cells, renal interstitial histiocytes | 3 cycles of CyBorD, combined with 2 cycles of BorD | PR， stable renal function(SCr0.74→1.25 mg/dl), improved Fanconi syndrome | 48 |

Abbreviation: M: Male; AKI: acute kidney injury; MGRS: monoclonal gammopathy of renal significance; CyBorD: cyclophosphamide, bortezomib, and dexamethasone; BorD: bortezomib and dexamethasone; VGPR: very good partial response; SCr: serum creatinine; F: Female; CKD: chronic kidney disease; NA: not available; eGFR: estimated Glomerular Filtration Rate; MGUS: monoclonal gammopathy of undetermined significance; PR : partial remission; CSH: crystal-storing histiocytosis; LCPT: light chain proximal tubulopathy

Referrences:

[S1] Qin A, Yu X, Zheng X et al. Lessons for the clinical nephrologist: a rare case with MGRS characterized by combined crystalline light chain proximal tubulopathy and crystal-storing histiocytosis responding to daratumumab. J Nephrol 2023; 36:1203-7.
[S2] Nakamura Y, Kitamura H, Ikai H et al. Combined light chain crystalline tubulopathy, podocytopathy, and histiocytosis associated with Bence–Jones κ protein diagnosed via immuno-electron microscopy. CEN Case Rep 2021; 10:453-8.
[S3] Ito K, Hara S, Yamada K et al. A case report of crystalline light chain inclusion-associated kidney disease affecting podocytes but without Fanconi syndrome: a clonal analysis of pathological monoclonal light chain. Medicine (Baltimore) 2019; 98: e13915.
